# Real-World Data Needs Real-World Doctors: When Automation Advances Faster Than Clinical Workflow

**DOI:** 10.2196/97972

**Published:** 2026-05-29

**Authors:** Amy Price, Christine Von Raesfeld

**Affiliations:** 1 Department of Community and Family Medicine (CFMED), Dartmouth-Hitchcock Clinics, Lebanon, NH, United States; 2 Journal of Participatory Medicine, JMIR Publications, Toronto, ON, Canada; 3 Center for Global Health, Colorado School of Public Health, University of Colorado Anschutz Medical Campus, 13001 E 17th Pl # B119, Aurora, CO, 80045, United States, 1 5618437372; 4 The Light Collective, Eugene, OR, United States

**Keywords:** artificial intelligence in medicine, clinical AI, real-world data, remote patient monitoring, digital health infrastructure, clinical decision support systems, diagnostic accuracy, clinical workflow integration, alert fatigue, physician burnout, workforce capacity, implementation science, care coordination, participatory medicine, coproduction of health care, patient-led research, health equity in AI, algorithmic governance, patient-centered care

## Abstract

Health care is entering an era of unprecedented detection. Artificial intelligence (AI)–driven monitors and real-world data streams now identify clinical risks in minutes, promising a future of proactive, earlier intervention. While AI automation is often marketed as a tool to reduce administrative burden and allow health care providers to focus more on direct patient care, this unrealized potential currently stands in contrast to our reality. Building high-speed data “freeways” without “off-ramps” such as clinical staffing, workflow synergy, and the patient education required for meaningful response is like building a superhighway without well-engineered off-ramps to provide a safe way to get home, and this creates a dangerous paradox. Earlier detection without earlier care does not improve outcomes; it simply redistributes anxiety and extends the patient’s period of uncertainty. We argue that the “public as a sensor” is already signaling a systemic infrastructure gap. True safety in clinical AI isn’t found in more algorithmic guardrails, but in participatory co-design that ensures every digital alert has a viable human pathway to care and resolution. We must stop building high-speed roads that lead to a cliff edge of clinical unavailability and consider that while the technology is a feat of engineering, it’s our human architecture that makes it medicine.

## Introduction

Health care systems can detect clinical problems earlier than at any point in history. Continuous cardiac monitors record rhythms for weeks, wearable devices capture subtle physiological changes throughout the day, and artificial intelligence (AI) algorithms analyze real-world data to identify patterns clinicians might otherwise miss [1-3]. Alerts generated by these systems move rapidly through electronic health records, often within minutes. The promise underlying this innovation is clear: earlier detection should lead to earlier intervention and better outcomes [4].

However, detection alone does not improve care [5]. Without a timely clinical response, earlier identification of risk can simply extend the period of uncertainty experienced by patients. This uncertainty is not neutral. It can be distressing, particularly when patients receive alerts suggesting potential clinical deterioration but cannot access timely evaluation.

**Figure 1.**
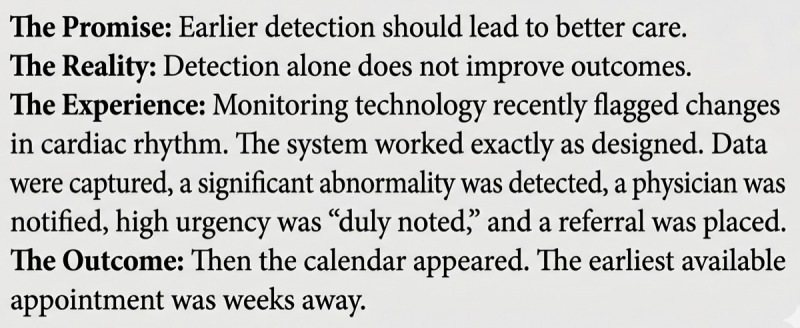
the When detection outpaces care: the patient's voice.

At the same time, discussions about clinical AI frequently emphasize guardrails designed to protect patients [6], while access to timely care remains constrained [7]. Patients experience a paradox: increased monitoring and earlier detection paired with delayed clinical response. In this context, safety narratives focused solely on algorithmic governance risk overlooking the lived experience of patients navigating uncertainty without timely clinical support [8]. This is detailed in Figure 1, which provides a narrative account of a patient encountering this systemic delay.

## Building Participatory Off-Ramps

Despite growing adoption of clinical AI, patients and the public are still rarely invited into meaningful partnerships to shape implementation, co-design workflows, or develop AI literacy initiatives. This absence is notable given that patients are often the first to experience the downstream consequences of a delayed response, increased alerting, and fragmented workflows [9].

Emerging research suggests that AI-enabled monitoring increases clinical signals and alerts, yet workflows and workforce capacity have not evolved at the same pace [10,11]. Without corresponding investment in response pathways, automation risks amplifying uncertainty rather than improving care.

This experience referenced in is increasingly common, even within large academic medical centers in the United States designed to provide rapid access to subspecialty expertise. In the US health care context, policy initiatives have encouraged the generation and use of real-world data. The issue is not technological capability alone, but whether health care systems have sufficient clinical capacity and workflow infrastructure to respond when new information emerges [12,13].

## The Paradox of Progress

Over the past decade, US policy initiatives have encouraged the generation and use of real-world data, including remote physiologic monitoring, patient-generated health data, and AI-supported triage systems. These technologies have expanded rapidly and demonstrated improved detection of deterioration across cardiovascular, metabolic, and chronic disease populations [4].

The engineering feats we are witnessing are nothing short of miraculous. We now have AI models identifying pancreatic cancer 3 years before a traditional diagnosis and wearable technology detecting silent heart failure from a wristband [14-16]. These are the high-speed lanes on the freeway of a new era in medicine.

To truly honor these breakthroughs, we must match this digital brilliance with an equally sophisticated human infrastructure. Our task is not to slow down the “freeway,” but to build the robust, graded “off-ramps,” the clinical time, the team-based staffing, and the participatory co-design that ensure every signal reaches its destination: a healthy patient. When we invite the public to coproduce technology as “sensors” and co-designers, we aren’t just fixing a workflow; we are building a health care system worthy of the technology we’ve created [17]. Table 1 shows the promise, reality, and proposed solutions.

Productive AI requires the meaningful involvement of patients and clinicians throughout the entire process, from co-design and ethics to defining alert thresholds, testing prototypes, assessing application quality, and shaping both feedforward concerns and feedback solutions, all while evaluating workflow integration. To ensure AI implementation truly serves those it is meant to help, we recommend fully compensated [18] “patient” and “clinician-in-the-loop” team roles, with genuine voting power over co-created outcomes.

Real-world data hold enormous promise for improving outcomes and personalizing care. They allow clinicians to observe disease trajectories in everyday settings and identify changes earlier than traditional episodic visits permit. Yet data alone do not improve health. Without clinicians available to interpret signals and systems capable of responding quickly, detection may simply shift the burden of waiting from before diagnosis to after notification and before timely intervention.

**Table 1.** Mapping the paradox of progress in clinical artificial intelligence (AI).

The promise	The reality gap	The participatory solution
Earlier detection	Alerts outpace workforce capacity. Detection happens in minutes; clinical response can take weeks.	Workflow co-design: involving patients and clinicians in prioritizing alerts and defining response expectations for high-quality health care.
Continuous monitoring	Increased patient anxiety. Earlier awareness without earlier care simply extends the period of uncertainty.	Human-centered guardrails: shifting focus from purely algorithmic governance to systemic safety, ethical agreement, and patient-supported partnership.
AI efficiency	Fragmented, “siloed” responses. Automation shifts effort toward interpretation and coordination, increasing cognitive demands.	Integrated care pathways: co-constructing response infrastructure, such as customer support and team-based care, as a primary part of deployment.

### Participatory Medicine Declaration

This work was co-produced, co-authored and pre–peer reviewed with patients.

## The Structure-Infrastructure Gap

**Figure 2.**
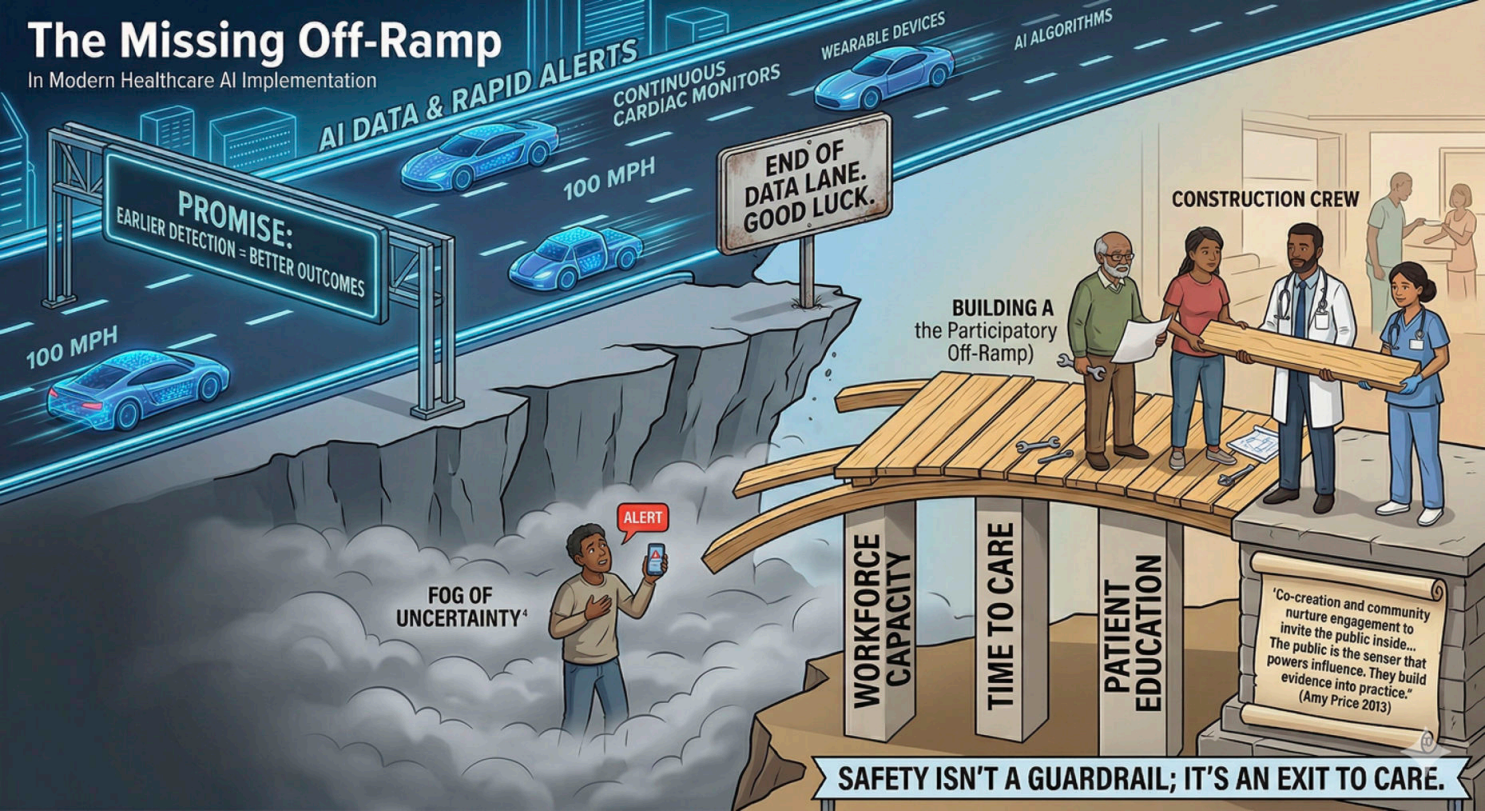
The missing off-ramp in modern health care artificial intelligence (AI) implementation. This conceptual illustration depicts the current disconnect between high-speed AI detection (the freeway) and the necessary systemic infrastructure required for clinical response (the off-ramp). Safe passage to high-quality health care requires bridging the gap between automated alerts and human-centered care through workforce capacity, protected clinical time, and participatory co-design. Image generated with the assistance of Google Gemini 3 Flash.

As emphasized above, we seek not to slow down the freeway, but to build off-ramps to ensure access to the destination: high-quality health care. Figure 2 illustrates the missing off-ramp for AI in health care.

AI automation is marketed as an efficient, cost-cutting solution to workforce shortages. Yet emerging evidence suggests a more complex reality. AI-enabled alerting systems, ambient documentation tools, and automated triage systems frequently introduce new cognitive demands, increase alert fatigue, and create additional coordination work for clinicians when workflows are not redesigned accordingly [19,20]. Rather than reducing workload, automation can shift effort from documentation to interpretation, triage, and coordination, tasks that still require complex and expert human clinical judgment [21].

## Workforce and Workflow Challenges

These workflow challenges occur in the context of worsening workforce shortages. Projections suggest significant physician shortages across primary care and subspecialties in the coming decade, further limiting the system’s ability to respond to newly detected risks. At the same time, structural barriers limit workforce expansion [22,23]. Medical school costs have risen substantially, creating financial barriers for many prospective physicians and influencing specialty choice toward higher-compensation fields, exacerbating shortages in primary care and community-based settings [24].

Training bottlenecks further constrain workforce growth. Residency and fellowship placements remain limited, meaning that even when medical school enrollment increases, postgraduate training capacity may not expand in proportion to the need [25]. This creates a structural mismatch between educational output and workforce needs, particularly as digital health technologies generate more clinical signals requiring interpretation [11].

Advanced practice clinicians, including nurse practitioners and physician associates, are increasingly expected to help address these shortages [26]. However, they often face a paradoxical situation: in some contexts, they are criticized for perceived gaps in expertise, while in others they are expected to manage increasingly complex patient populations and respond to AI-generated alerts beyond traditional scope-of-practice boundaries [27]. Evidence suggests that team-based care models improve outcomes when roles are clearly defined and supported, rather than when advanced practice clinicians are used as substitutes for unavailable physicians [27].

For patients, this gap shifts responsibility in subtle but important ways. When notified of abnormal findings, individuals often monitor symptoms more closely, search for information, and attempt to interpret data while waiting for professional guidance [13]. For those living with chronic or complex conditions, this period can be particularly challenging. Instead of reducing uncertainty, earlier detection may extend it. In this sense, automation without response capacity risks creating a new form of harm: early awareness without early care [13,28].

## Conclusion: Toward a Sociotechnical Solution

Emerging informatics and implementation science emphasizes that successful digital transformation requires co-design with clinicians and patients, workflow redesign, and evaluation of downstream response capacity, not simply detection accuracy [7,29]. These findings reinforce the view that AI implementation is fundamentally a sociotechnical challenge rather than a purely technological one.

The next phase of digital health policy must move beyond generating data and toward ensuring that care systems can act on the information they produce. Praising automation while neglecting clinical workflow and workforce capacity risks creating unintended harm. Real-world data will only transform health care if real-world doctors, and the teams who support them, are available to respond.

We have invested billions in data infrastructure, that is, the high-speed lanes, while neglecting the off-ramps that reduce mortality and increase safety: the clinicians, time to task, patient education, and cost required to transition from a digital alert to a clinical resolution. As highlighted in Figure 2, safety is not just a guardrail; it means ensuring there is a safe way to get home.

## Supplementary material

10.2196/97972 Multimedia Appendix 1 Prompt used in image generation.

## Abbreviations

AI: artificial intelligence
