# Supplementary material for: Real-World Data Needs Real-World Doctors: When Automation Advances Faster Than Clinical Workflow
Source: J Particip Med. 2026 May 29;18:e97972. doi: 10.2196/97972 (PMC13221120; doi:10.2196/97972)
Supplement: Multimedia Appendix 1 [file jopm-v18-e97972-s001.docx]

## We used Gemini Flash 3 to generate Figure 1 with our prompt methods: Based on discussions, the prompt was built around these specific visual metaphors:

- **The Data Freeway:** A multi-lane, high-speed highway representing "AI Data & Rapid Alerts" with cars labeled "Continuous Cardiac Monitors," "Wearable Devices," and "AI Algorithms".
- **The Cliff-Edge:** The "End of Data Lane," where the high-speed road ends abruptly at a precipice.
- **The Fog of Uncertainty:** A valley below the cliff where a patient is standing, holding a phone with a red "Alert" notification.
- **The Participatory Off-Ramp:** A sturdy bridge or "off-ramp" being constructed to span the gap.
- **The Construction Crew:** A collaborative team of patients and clinicians (including doctors and nurses) physically building the bridge with wooden planks.
- **The Foundation Pillars:** The bridge is supported by pillars labeled **Workforce Capacity**, **Time to Care**, and **Patient Education**.

**Prompt**: "A professional, clean medical infographic titled 'The Missing Off-Ramp.' A high-speed digital freeway representing healthcare AI alerts ends at a sharp cliff-edge labeled 'End of Data Lane.' Below, a patient stands in a 'Fog of Uncertainty' looking at a phone alert. A diverse construction crew of clinicians and patients is building a sturdy wooden bridge (the Off-Ramp) across the chasm. The bridge is supported by three massive stone pillars labeled 'Workforce Capacity,' 'Time to Care,' and 'Patient Education.' Style: modern, educational, conceptual illustration."

Gemini Flash suggested putting a quote from our article in the graphic. We liked it and accepted the image.
